# ADCC: An underappreciated correlate of cross-protection against influenza?

**DOI:** 10.3389/fimmu.2023.1130725

**Published:** 2023-02-22

**Authors:** Rory D. de Vries, Katja Hoschler, Guus F. Rimmelzwaan

**Affiliations:** ^1^ Department of Viroscience, Erasmus Medical Center, Rotterdam, Netherlands; ^2^ Virus Reference Department, Public Health England, London, United Kingdom; ^3^ Research Center for Emerging Infections and Zoonoses, University of Veterinary Medicine, Hannover, Germany

**Keywords:** influenza virus, vaccination, infection, antibodies, ADCC

## Abstract

In this short review, we summarized the results obtained with an assay to detect influenza virus-specific antibodies that mediate ADCC, which was developed and evaluated within the framework of the IMI-funded project “FLUCOP”. HA-specific ADCC mediating antibodies were detected in serum samples from children and adults pre- and post-vaccination with monovalent, trivalent, or quadrivalent seasonal influenza vaccines, or following infection with H1N1pdm09 virus. Additionally, using chimeric influenza HA proteins, the presence of HA-stalk-specific ADCC mediating antibodies after vaccination and natural infection with H1N1pdm09 virus was demonstrated. With serum samples obtained from children that experienced a primary infection with an influenza B virus, we showed that primary infection induces HA-specific ADCC-mediating antibodies that cross-reacted with HA from influenza B viruses from the heterologous lineage. These cross-reactive antibodies were found to be directed to the HA stalk region. Antibodies directed to the influenza B virus HA head mediated low levels of ADCC. Finally, vaccination with a recombinant modified vaccinia virus Ankara expressing the HA gene of a clade 1 A(H5N1) highly pathogenic avian influenza virus led to the induction of ADCC-mediating antibodies, which cross-reacted with H5 viruses of antigenically distinct clades. Taken together, it is clear that virus-specific antibodies induced by infection or vaccination have immunological functionalities in addition to neutralization. These functionalities could contribute to protective immunity. The functional profiling of vaccine-induced antibodies may provide further insight into the effector functions of virus-specific antibodies and their contribution to virus-specific immunity.

## 1 Introduction

Influenza viruses cause seasonal outbreaks of respiratory disease associated with substantial morbidity and mortality. These outbreaks of influenza are caused by influenza A viruses (IAV) of the H1N1 and H3N2 subtypes, and influenza B virus (IBV). Two lineages of IBV have been distinguished that have cocirculated since the 1970s, the B/Victoria/2/87-like (B/Vic) lineage and the B/Yamagata/16/88-like (B/Yam) lineage, which differ based on genetic and antigenic properties of their hemagglutinins (1, 2). Since the start of the coronavirus disease-2019 (COVID-19) pandemic, IBV of the B/Yam lineage has hardly been detected, and likely was eliminated (3).

Vaccines are available to protect against seasonal influenza and especially high-risk patients benefit from annual vaccination. However, the design of effective vaccines is complicated by the accumulation of antigenic changes in the viral protein hemagglutinin (HA) by seasonal influenza viruses, which is known as antigenic drift. Because HA is the main target for the development of virus-neutralizing (VN) antibodies and the major component of influenza vaccines, the composition of the vaccines needs to be updated almost annually to guarantee optimal effectiveness (4). The currently used seasonal influenza vaccines are quadrivalent and contain components of IAV subtypes H1N1 and H3N2, as well as both IBV lineages. However, the potential elimination of IBV from the B/Yam lineage could have implications for future vaccine design.

Upon influenza virus infection, virus-specific antibody and T cell responses are induced. Virus-neutralizing antibodies directed to the viral HA are considered an important correlate of protection from infection, provided that they match the influenza virus causing the infection antigenically. For the detection and quantification of virus-specific serum antibodies the hemagglutination inhibition (HI) and VN assays are commonly used. HI antibody titers are an accepted proxy for VN antibodies and are used to determine the efficacy of seasonal influenza vaccines. HI antibody titers are considered a good correlate of protection (5), although the HI assay suffers from some disadvantages, like the difficulty to standardize. Furthermore, the HI assay is not suitable for the detection of antibodies directed to antigenic regions of the HA molecule located outside the receptor-binding domain (RBD), like HA-stalk-specific antibodies that are currently of interest for universal influenza vaccine designs (6).

VN antibodies are typically directed to epitopes located in and around the RBD of the variable globular head domain of HA and thus can block binding of the virus to its receptor on host cells. Most HI antibodies are strain-specific and do not recognize variants that have accumulated one or more amino acid substitution in vicinity of the RBD (2). Because the head domain is immunodominant, the majority of antibodies to HA, induced after natural infection or vaccination, is directed to the head. However, antibodies to the more conserved HA-stalk are induced as well. These antibodies can neutralize influenza virus *via* a different mechanism than preventing receptor interaction, namely by preventing conformational changes of HA that take place in the endosome in a pH dependent fashion. These antibodies additionally exert other biological activities that contribute to protective immunity, which are described below. Because the HA-stalk is relatively conserved, even across various subtypes of IAV, stalk-specific antibodies are highly cross-reactive.

Antibodies also have biological activities other than virus neutralization, like antibody-dependent phagocytosis (ADP), antibody-dependent complement deposition (ADC) and antibody dependent cellular cytotoxicity (ADCC). All of these activities are mediated *via* Fc-receptors and contribute to protective antiviral immunity, as was shown for influenza virus (7–9), human respiratory syncytial virus (HRSV) (10–12), and severe acute respiratory distress syndrome coronavirus-2 (SARS-CoV-2) (13–15). The induction of antibodies to the conserved HA stalk and their mode of action is important for the development of broadly protective influenza vaccines. ADCC takes place when antibodies bind to proteins that are expressed on the surface of virus-infected cells (like HA). When NK cells or neutrophils engage with these antibodies through binding of the Fc part of an antibody with their Fcγ-receptor IIIa (FcγRIIIa, CD16), they become activated and can kill infected cells through the release of lytic granules. For the detection of ADCC-mediating serum antibodies induced after vaccination with existing or novel candidate universal influenza, the availability of reliable assays, suitable for standardization, is required.

In the framework of the Innovative Medicines Initiative (IMI) funded project “FLUCOP”, which aimed at the standardization and development of assays for assessment of influenza vaccines correlates of protection, we developed and used an assay for the detection of antibodies with ADCC activity that would be suitable for standardization. Here, we compiled antibody-dependent cellular cytotoxicity (ADCC) data obtained in four separate studies to get a comprehensive overview of the induction of ADCC-mediating antibodies after influenza virus infection, or immunization with monovalent, trivalent, or quadrivalent influenza vaccines (16–19).

## 2 Methods

### 2.1 Ethics statement and serum panels

All data shown here were obtained with serum samples from subjects that were previously enrolled in other studies; ethical permission was therefore previously obtained.

Serum samples were obtained from 9- and 10-year-old children before and after infection with the H1N1pdm09 virus (Trial ISRCTN64117538) (20). In addition, five serum pairs from subjects that displayed seroconversion, were obtained from other studies (Cohort IK) (21–23). Serum samples were collected from laboratory-confirmed H1N1pdm09 cases on the day of clinical onset and up to three subsequent time points. This study was approved by the Medical Ethical Review Committee of the University Medical Centre Utrecht (Cohort CF) (22, 24). Serum samples were obtained from forty-five unvaccinated children 1–7 years of age who experienced a primary infection with influenza B virus of the B/Vic lineage (N=21) or the B7Yam-lineage (N=24) (Cohort Pienter) (25, 26). Serum samples were obtained from health care workers before vaccination with the H1N1pdm09 MF59-adjuvanted vaccine (Focetria, Novartis) three weeks later and five weeks after a booster vaccination (Cohort Tilburg) (27). Serum samples were obtained from 44 study subjects before and 4 and 8 weeks after receiving a recombinant MVA encoding the HA gene of influenza virus A/Vietnam/1194/04 (H5N1. In addition, serum samples were collected from 27 of these study subjects before and 4 and 20 weeks after receiving a booster vaccination after one year (Cohort MVA-H5) (28). Serum samples were obtained from healthy young study subjects before and 7 days after receiving an adjuvanted seasonal trivalent vaccine (Cohort FLUAD). Paired sera were obtained from 42 healthy study subjects, of which 27 received TIV with an influenza B component of the B/Yam lineage only and 15 received QIV with influenza B components of both the B/Yam and B/Vic lineages REF (Cohort GRC). Serum samples were obtained from 20 study subjects before and 21 days after receiving the Live-attenuated influenza vaccine (LAIV) Fluenz (Cohort PHE).

The serum panels included in this study are summarized in **Table 1**. All work described has been carried out in accordance with the code of ethics of the world medical association (declaration of Helsinki).

**Table 1:** Compilation of all serum sets used in the present study. In this compilation, serum samples from 10 different cohorts were included.

exposure	cohort	age	N	cohort	antigen	timepoints	reference
infection	pH1 primary infection	children	5	IK	pH1N1 infection	pre and post infection	(18)
infection	pH1 infection	adults	22	CF	pH1N1 infection	during and post infection	(18)
infection	B/Vic primary infection	children	21	Pienter	B/Vic infection	single timepoint	(19)
infection	B/Yam primary infection	children	24	Pienter	B/Yam infection	single timepoint	(19)
vaccination	monovalent H1	adults	22	Tilburg	H1	pre and post vaccination	(18)
vaccination	monovalent H5	adults	44	MVA-H5	H5	pre and post vaccination	(16)
vaccination	trivalent	adults	27	GRC	H1, H3, B/Yam	pre and post vaccination	(17)
vaccination	trivalent	adults	24	FLUAD	H1, H3, B/Yam	pre and post vaccination	(18)
vaccination	quadrivalent	adults	15	GRC	H1, H3, B/Yam, B/Vic	pre and post vaccination	(17)
vaccination	quadrivalent (LAIV)	unknown	20	PHE	H1, H3, B/Yam, B/Vic	pre and post vaccination	–

As for infection samples, sera were obtained from children pre- and post-H1N1pdm09 virus infection (N=5, IK), adults acutely infected with H1N1pdm09 virus (N=22, CF), and children that experienced a primary infection with B/Vic (N=21) or B/Yam (N=24, both Pienter). As for vaccination samples, sera were obtained from health care workers vaccinated with monovalent adjuvanted H1N1pdm09 vaccine (N=22, Tilburg), monovalent vector-based H5 vaccine (N=44, MVA-H5), adults that received adjuvanted seasonal trivalent inactivated vaccine (N=27, GRC and N=24, FLUAD), and adults that received adjuvanted seasonal quadrivalent vaccine (N=15, GRC). A complete description of the cohorts can be found in (16–19).

### 2.2 Antibody-dependent NK cell activation assay

For the detection of serum antibodies mediating influenza virus-specific ADCC activity, we used the human NK cell line NK92.05-CD16 that expresses the FcγRIII receptor (CD16) as previously described (18). NK92.05-CD16 cell stocks tested negative for contamination with *Mycoplasma* species, and were only cultured for a limited number of passages. In brief, microtiter plates were coated with recombinant protein (HA, chimeric HA or NA) and after blocking the plates with bovine serum albumin (BSA), specific serum antibodies were allowed to bind at a serum dilution of 1:160. This dilution was found to be optimal and in the linear range of the dose response curve. A serum pool from uninfected children, intravenous immunoglobulins (IVIG, Kiovig or Privigen) and a high-positive serum were included in the experiments as negative and positive controls. After the serum incubation, 1x10^(5)^ NK92.05-CD16 cells were added per well in the presence of a monoclonal antibody to CD107a, (degranulation marker), golgistop and golgiplug. After incubation for 5 hours at 37°C, the cells were stained for flow cytometric analysis. Live NK92.05-CD16 cells were identified by LIVE/DEAD and CD56 staining as described previously; CD56-PE (BD Biosciences) was used in a concentration of 1µg/ml, aqua live/dead (Thermo Fisher) was used per manufacturer’s instruction (14, 18). Subsequently, cells were fixed and analysed by flow cytometry and the proportion of responding, CD107a-positive cells, was determined.

### 2.3 Antigens

For the detection of ADCC-mediating antibodies to influenza A and B viruses, several antigens were used as previously described (16–19). Purified full-length baculovirus expressed recombinant HA derived from influenza viruses A/Cal/07/09 [pH1], A/Texas/50/12 [H3], B/Brisbane/60/08 [B/Vic], B/Malaysia/2506/04 [B/Vic-HA1], B/Yamanashi/166/98 [B/Yam], B/Florida/4/06 [B/Yam-HA1], A/Vietnam/1194/04 [H5-VN], or A/Indonesia/5/05 [H5-IND] were used in ADCC assays (made available by Protein Sciences or kind gift from Florian Krammer). For the detection of antibodies to the HA stalk, chimeric full-length antigens were used. For the detection of antibodies to the stalk of influenza B viruses, a chimeric HA was used consisting of the head domain of an avian IAV of the H8 subtype (A/mallard/Sweden/24/02) and the stalk of IBV B/Yamagata/16/88 (cH8/B) (kind gift from Florian Krammer).

### 2.4 Statistical analysis

Differences between two groups were assessed by either Mann-Whitney U test or Wilcoxon rank test, dependent on whether unpaired or paired samples were used. Differences between multiple groups were assessed by Kruskal-Wallis test with multiple comparisons. Linear regression on log-transformed ADNK activation percentages was performed for the serum samples from MVA-H5 vaccinated participants.

## 3 Results

### 3.1 Cohort description

To get a complete overview of the induction of ADCC-mediating antibodies by influenza virus infection or influenza vaccination, we compiled datasets from four different studies (**Table 1**). These studies contained a total of 10 different cohorts; in 4 cohorts, samples were obtained before and after influenza virus infection. In 6 cohorts, samples were obtained before and after influenza vaccination. The infection cohorts consisted of both immunologically naive children as well as adults with pre-existing immunity. The vaccination cohorts consisted exclusively of adults; however, both the primary induction of ADCC-mediating antibodies as well as the immunological recall was studied by using antigens to which pre-existing immune responses were absent (H5) or present (H1, H3, B).

### 3.2 Induction of ADCC-mediating antibodies by primary infection

**Figure 1:**
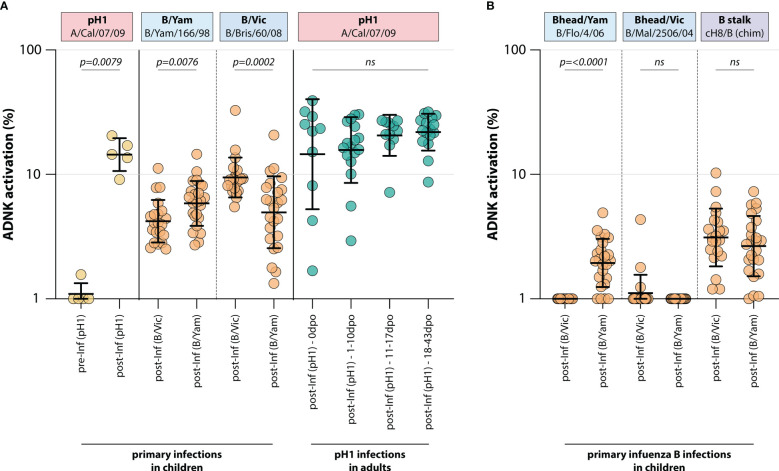
Induction of ADCC-mediating antibodies after H1N1pdm09, B/Vic or B/Yam primary infection. **(A)** levels of ADCC-mediating antibodies in infected children or adults measured with full-length HA proteins. ADCC-mediating antibodies were determined in H1N1pdm09 infected children, who were H1N1pdm09 seronegative prior to 2009 and seroconverted (yellow). Additionally, ADCC-mediating antibodies were determined in B/Vic or B/Yam infected children in sera obtained from a surveillance study (orange). ADCC-mediating antibodies were also measured in H1N1pdm09 virus infected adults from the timepoint of clinical onset and onwards (green). **(B)** Levels of ADCC-mediating in B/Vic or B/Yam infected children measured with ‘head only’ HA proteins, or a chimeric HA protein with the H8 head and influenza B virus stalk (cH8/B) (orange). ADCC was measured in identical sera to panel **(A)** Serum cohorts and timepoints are indicated on the x-axis, proteins against which ADCC was assessed are indicated in boxes above the graph. Full-length HA proteins were used unless indicated otherwise. ADNK, antibody-dependent natural killer; Inf, infection; dpo, days post onset; ns, non-significant. Differences between two groups were assessed by Mann-Whitney U test, differences between multiple groups were assessed by Kruskal-Wallis test with multiple comparisons.

To determine whether a primary infection with influenza virus induced ADCC-mediating antibodies, ADCC was measured in a unique sample set of sera from 5 children obtained before and after primary infection with H1N1pdm09 influenza virus (IK cohort, **Table 1**). As expected, pH1-specific ADCC-mediating antibodies could not be detected prior to infection, whereas all children had ADCC-mediating antibodies post-infection (**Figure 1**).

Similarly, ADCC-mediating antibodies were measured in 45 children after a primary infection with IBV of either the B/Vic or the B/Yam lineage (Pienter cohort, **Table 1**). Although serum pre-infection was not available, a preferential reactivity with the homologous IBV HA protein was observed post-infection. Children infected with B/Vic had relatively more ADCC reactivity with the B/Vic HA protein, and vice versa (**Figure 1**). Interestingly, cross-reactivity with the HA protein from the other IBV lineage was observed only when full-length HA proteins were used. When the ADCC assay was performed with ‘head-only’ antigens, homologous reactivity was exclusively observed (albeit at low level) and cross-reactivity with the other lineage was lost (**Figure 1**). Both B/Vic and B/Yam infection induced ADCC-mediating antibodies targeting the IBV stalk, suggesting that cross-reactivity is mediated by stalk-specific antibodies (**Figure 1**). Finally, the presence of pH1-specific ADCC-mediating antibodies was measured in adults experiencing a H1N1pdm09 virus infection at 4 different timepoints: 0 days post onset symptoms (dpo, first symptomatic day), 1-10 dpo, 11-17 dpo, and 18-43 dpo. In these sera, ADCC-mediating antibodies were already present at 0dpo (could be pre-existing, or triggered by this infection), and no significant increase was measured over time (**Figure 1**).

### 3.3 Induction of ADCC-mediating antibodies with monovalent vaccines

**Figure 2:**
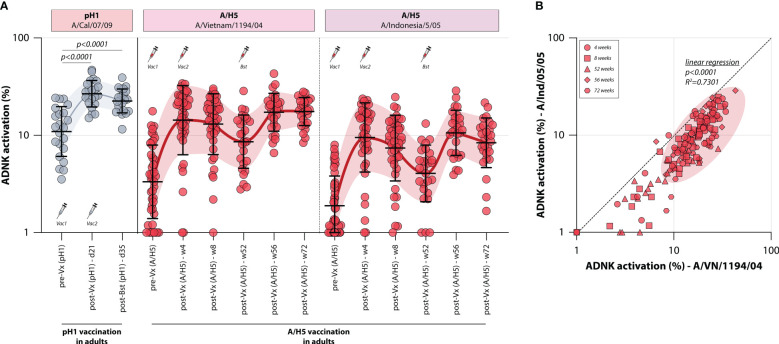
Induction of ADCC-mediating antibodies after monovalent H1N1pdm09 or MVA-H5 vaccination. **(A)** levels of ADCC-mediating antibodies in vaccinated adults measured with full-length HA proteins. ADCC-mediating antibodies targeting the pH1 protein were determined in H1N1pdm09 vaccinated adults, who proved seropositive prior to vaccination (indicative of pre-existing immunity) (gray). Additionally, ADCC-mediating antibodies were measured in monovalent MVA-H5 vaccinated adults (red). ADCC reactivity to both the homologous H5 (A/Vietnam/1194/04) as a heterologous H5 protein (A/Indonesia/5/05) was determined. Serum cohorts and timepoints are indicated on the x-axis, proteins against which ADCC was assessed are indicated in boxes above the graph. Full-length HA proteins were used unless indicated otherwise. **(B)** Correlation between ADCC levels in sera from MVA-H5 vaccinated adults against the homologous or heterologous H5 protein. Symbols indicate different timepoints, diagonal line is a reference to reflect equal levels to the homologous and heterologous proteins. ADNK, antibody-dependent natural killer; Vx, vaccination; w, week. Differences between two groups were assessed by Mann-Whitney U test. Linear regression on log-transformed ADNK activation percentages was performed.

To assess whether influenza vaccines recall or induce ADCC-mediating antibodies, the levels of these antibodies were measured in two different monovalent vaccination cohorts. First, ADCC was measured in a sample set of sera from 22 adults obtained before and at two timepoints after vaccination with a monovalent H1N1pdm09 vaccine (Tilburg cohort, **Table 1**). It was observed that the adults included in this trial had pre-existing pH1-specific ADCC-mediating antibodies, but monovalent vaccination led to a rapid increase of these antibodies targeting the homologous HA on day 21 post vaccination (**Figure 2**). A further increase towards 35 days post vaccination was not observed.

Similarly, the induction of ADCC-mediating antibodies by vaccination with a novel antigen was determined. To this end, 44 adults were vaccinated with a vector-based H5 vaccine (MVA-H5 cohort, **Table 1**). Participants received 1 or 2 shots of MVA-H5 (at a dose of 10^7^ or 10^8^ plaque forming units), and selected study subjects were boosted with an additional vaccine 1 year later. Serum samples were obtained prior to vaccination and throughout the trial. ADCC-mediating antibodies targeting the full-length homologous H5 protein (A/Vietnam/1194/04) were detected prior to vaccination in some participants, although exposure to an H5 IAV was not deemed likely (**Figure 2**). ADCC-mediating antibodies increased rapidly at 4 weeks post vaccination, a further increase at 8 weeks post vaccination was not observed. After that, antibodies waned until the booster vaccination was given one year later, rapidly recalling H5-specific ADCC-mediating antibodies. After booster, the levels remained stable up to 20 weeks post-booster vaccination. The ADCC-mediating antibodies proved cross-reactive with a heterologous H5, as similar induction, waning and recall patterns were observed when sera were tested against this full-length HA of a virus belonging to an antigenically distinct clade of H5 viruses (A/Indonesia/5/05) (**Figure 2**). However, a direct correlation between the two antigens showed that sera were more reactive to the homologous antigen that was included in the vaccine, compared to the heterologous antigen (**Figure 2**).

### 3.4 Induction of ADCC-mediating antibodies by seasonal influenzas vaccines

**Figure 3:**
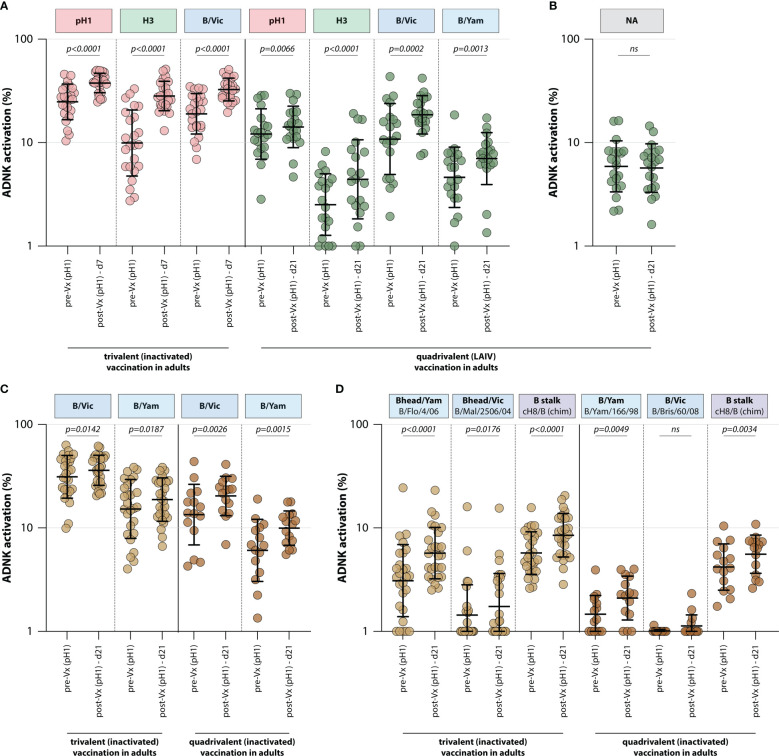
Induction of ADCC-mediating antibodies after seasonal influenza vaccination. **(A)** Levels of ADCC-mediating antibodies in vaccinated adults measured with full-length HA proteins. ADCC-mediating antibodies targeting the pH1, H3, B/Vic and B/Yam HA proteins were determined in inactivated trivalent (red) or live-attenuated quadrivalent (green) adult vaccine recipients. **(B)** Levels of neuraminidase (NA)-specific ADCC-mediating antibodies in the live-attenuated quadrivalent adult vaccine recipients. **(C)** Specific focus on B/Vic or B/Yam HA-specific ADCC-mediating antibodies in adults receiving inactivated trivalent (yellow) or quadrivalent (orange) seasonal influenza vaccine. **(D)** Levels of ADCC-mediating in samples shown in **(C)** with ‘head only’ HA proteins, or a chimeric HA protein with the H8 head and influenza B virus stalk. Serum cohorts and timepoints are indicated on the x-axis, proteins against which ADCC was assessed are indicated in boxes above the graph. Full-length HA proteins were used unless indicated otherwise. ADNK, antibody-dependent natural killer; Vx, vaccination; d, day; LAIV, live-attenuated influenza vaccine. Differences between two groups were assessed by Wilcoxon rank test.

To assess whether trivalent or quadrivalent influenza vaccines recall ADCC-mediating antibodies, the levels of these antibodies were measured in different adult vaccination cohorts. ADCC-mediating antibodies targeting the IAV pH1 and H3 proteins were measured, as well as those targeting the IBV HA protein (homologous to the protein included in the vaccine). By measuring ADCC-mediating antibodies after adjuvanted inactivated trivalent vaccination in 24 adults (FLUAD cohort, **Table 1**), it was shown that vaccination boosts ADCC-mediating antibodies. Antibody levels against all antigens included in the vaccine (pH1, H3, and B/Vic) significantly increased at 7 days post vaccination (**Figure 3**). Similarly, quadrivalent vaccination with a live-attenuated influenza vaccine boosted ADCC-mediating antibodies targeting all proteins included in the vaccine at 21 days post vaccination (pH1, H3, B/Vic, and B/Yam, **Figure 3**). In these same sera ADCC-mediating antibodies targeting the NA protein were measured. Although these antibodies could be detected prior to vaccination, a boost in NA-specific ADCC was not observed (**Figure 3**).

Finally, the effect of trivalent and quadrivalent inactivated vaccination on ADCC-mediating antibodies targeting the full-length HA from influenza B/Vic and B/Yam virus was measured in 42 adults (N=27 trivalent vaccinated, N=15 quadrivalent vaccinated, GRC cohorts, **Table 1**). Interestingly, an increase in ADCC-mediating antibodies targeting B/Vic and B/Yam was observed in participants that received either the trivalent or quadrivalent vaccine, even though the trivalent vaccine only included a B/Vic component (**Figure 3**). In addition to an increase in ‘head-only’-specific ADCC-mediating antibodies, IBV stalk-specific antibodies increased in both cohorts at 21 days post vaccination (**Figure 3**), indicative of boosting of cross-reactive antibodies targeting conserved epitopes.

## 4 Concluding remarks

In this brief review, we have summarized findings on the induction of ADCC antibodies by influenza virus infection, or by vaccination with monovalent, trivalent, or quadrivalent seasonal and pandemic influenza vaccines. For the detection of ADCC-mediating antibodies we developed an assay using an NK cell line that expresses the FcγRIIIa receptor (CD16) in combination with recombinant proteins bound to a solid phase. Activation of NK cells *via* antibodies that bound the antigens of interest was subsequently measured by assessing CD107a expression. Using this assay and serum samples from selected well-documented human serum panels, obtained from participants that received various vaccines or that experienced infections with influenza A or B viruses, the induction of ADCC mediating antibodies was measured. As serum was used, this assay did not differentiate between the biological activity of the different Ig subclasses. Additionally, due to the retrospective nature of this study and privacy legislation, we were unable to acquire potentially relevant metadata like age and biological sex. Although the evidence is limited, there are previous reports that show a difference in induction of ADCC-mediating antibodies between male and females after measles vaccination (29). The use of native and chimeric HA molecules in the assay allowed distinction of antibody reactivity with the entire HA, HA head, or the stalk region of HA only. Our assays showed that antibodies targeting the relatively conserved stalk region of the HA molecule could mediate ADCC, which was also found by other research groups (7, 8, 30).

We developed this assay in the framework of the IMI project “FLUCOP”, aimed at the standardization and development of assays for assessment of influenza vaccines correlates of protection. Although we found that results obtained with the developed ADCC degranulation assay were highly reproducible, the assay still depended on biological components (NK cells) and is relatively low-throughput. For that reason, reporter assays based on cells that express luciferase upon engagement of FcγRIIIa have been developed. Recently, results obtained with the degranulation assay were compared with those obtained with a reporter assay, which showed that the results strongly and significantly correlated (31). The reporter assay might be a good substitute for the degranulation assay, especially in settings where larger sample numbers need to be analyzed. Although the use of reporter cells may be more suitable for high throughput testing and easier to standardize than flowcytometric analysis of CD107a expression, there may still be a need for true functional assays with relevant cells.

It is clear that virus-specific antibodies, induced by either infection or vaccination, have immunologically relevant functionalities, in addition to neutralization of virus infectivity by blocking glycoprotein – receptor interactions; ADP-, ADC- and ADCC-mediating antibodies are likely to contribute to protective immunity (7, 8, 10–15). Therefore, functional profiling of vaccine-induced antibodies in clinical trials is crucial to provide further insight into Fc-mediated effector functions of virus-specific antibodies and their contribution to virus-specific immunity. In addition to functional profiling, it is important to study IgG subclasses induced after vaccination or infection. Indeed, the IgG subclasses interact differently with Fcγ-receptors (IgG2 and IgG4 for example display limited interaction with FcγRIIIa or CD16) and post-translational processes like fucosylation can dampen the affinity of antibodies for Fcγ-receptors (11).

The importance of functionality profiling was recently demonstrated in a phase I clinical trial, in which chimeric hemagglutinin-based universal influenza virus vaccines were used to induce broad immunity. This was achieved by targeted boosting of HA-stalk-specific antibodies; because the HA stalk is conserved, these antibodies contribute to heterosubtypic immunity. In this trial, not only did the authors demonstrate the presence of broadly-reactive stalk-specific antibodies, they also showed functionality in neuraminidase inhibition and ADCC reporter assays. Passive transfer of these antibodies in mice showed that they were indeed an independent correlate of protection (9). The induction of ADCC-mediating antibodies to conserved epitopes is therefore considered a promising target for the development of vaccines that afford broadly protective immunity.

Together, we showed that influenza virus infection and influenza vaccination led to the induction of ADCC-mediating antibodies, which can be rapidly recalled by re-infection or re-vaccination. ADCC-mediating antibodies often target epitopes in the relatively conserved HA-stalk, potentially important to achieve broadly-reactive immunity. We conclude that complete functional profiling of the antibody responses is crucial to provide insight into effector functions of virus-specific antibodies and their contribution to virus-specific immunity. In addition to profiling antibody responses in blood, it is important to additionally assess the contribution of IgA at the mucosal sites. The availability of reliable assays for the measurement of functional antibodies will aid the evaluation of existing and novel influenza vaccines.

## Author contributions

RDdV and GFR conceptualized the manuscript. KH organised clinical samples for the study. All authors contributed to the writing of the manuscript. KH contributed to the study design.
